# Supplementary material for: Large-Scale Profiling of Extracellular Vesicles Identified miR-625-5p as a Novel Biomarker of Immunotherapy Response in Advanced Non-Small-Cell Lung Cancer Patients
Source: Cancers (Basel). 2022 May 14;14(10):2435. doi: 10.3390/cancers14102435 (PMC9139420; doi:10.3390/cancers14102435)
Supplement: Supplementary file 1 [file cancers-14-02435-s001.zip › Supplementary Table S1.pdf]

Dependent : Objective Response		Not Responder	Responder	OR (univariable)
**Setting**	First Line	19 (51.4%)	18 (48.6%)	-
	Second or Third Line	45 (88.2%)	6 (11.8%)	0.14 (0.04-0.39, p= < 0.001)
**Treatment**	Pembrolizumab	30 (56.6%)	23 (43.4%)	-
	Nivolumab	34 (97.1%)	1 (2.9%)	0.04 (0.00-0.20, p= 0.002)
**PDL1 Staning**	≥50%	23 (52.3%)	21 (47.7%)	-
	<50%	41 (93.2%)	3 (6.8%)	0.08 (0.02-0.26, p < 0.001)
**hsa.miR.625.5p**	Low	14 (42.4%)	19 (57.6%)	-
	High	50 (90.9%)	5 (9.1%)	0.07 (0.02-0.22, p < 0.001)
