# Supplementary material for: Large-Scale Profiling of Extracellular Vesicles Identified miR-625-5p as a Novel Biomarker of Immunotherapy Response in Advanced Non-Small-Cell Lung Cancer Patients
Source: Cancers (Basel). 2022 May 14;14(10):2435. doi: 10.3390/cancers14102435 (PMC9139420; doi:10.3390/cancers14102435)
Supplement: Supplementary file 1 [file cancers-14-02435-s001.zip › Supplementary Table S2.pdf]

Dependent : Overall Survival		All	HR (univariable)
**Setting**	First Line	37 (42.0%)	-
	Second or Third Line	51 (58.0%)	1.75 (1.07-2.85, p= 0.025)
**Treatment**	Pembrolizumab	53 (60.2%)	-
	Nivolumab	35 (39.8%)	1.97 (1.22-3.16, p= 0.005)
**ECOG**	0	40 (45.5%)	-
	1	48 (54.5%)	1.65 (1.01-2.69, p= 0.044)
**Tumor Burden**	≥ 115 mm	46 (52.3%)	-
	< 115 mm	42 (47.7%)	0.51 (0.31-0.82, p= 0.005)
**Liver Metastases**	Yes	71 (80.7%)	-
	No	17 (19.3%)	2.69 (1.50-4.80, p= 0.001)
**PDL1 Staning**	≥50%	44 (50.0%)	-
	<50%	44 (50.0%)	2.87 (1.75-4.72, p < 0.001)
**hsa.miR.625.5p**	Low	33 (37.5%)	-
	High	55 (62.5%)	2.14 (1.28-3.58, p= 0.004)
