# Supplementary material for: Large-Scale Profiling of Extracellular Vesicles Identified miR-625-5p as a Novel Biomarker of Immunotherapy Response in Advanced Non-Small-Cell Lung Cancer Patients
Source: Cancers (Basel). 2022 May 14;14(10):2435. doi: 10.3390/cancers14102435 (PMC9139420; doi:10.3390/cancers14102435)
Supplement: Supplementary file 1 [file cancers-14-02435-s001.zip › Supplementary Table S3.pdf]

| Dependent : Progression Free Survival | | All | HR (univariable) |
|---|---|---|---|
| **Setting** | First Line | 37 (42.0%) | - |
| | Second or Third Line | 51 (58.0%) | 1.80 (1.11-2.91, p= 0.016) |
| **Treatment** | Pembrolizumab | 53 (60.2%) | - |
| | Nivolumab | 35 (39.8%) | 1.68 (1.05-2.69, p= 0.031) |
| **Tumor Burden** | ≥ 115 mm | 46 (52.3%) | - |
| | < 115 mm | 42 (47.7%) | 0.49 (0.31-0.78, p= 0.003) |
| **Liver Metastases** | Yes | 71 (80.7%) | - |
| | No | 17 (19.3%) | 2.45 (1.38-4.37, p= 0.002) |
| **PDL1 Staning** | ≥50% | 44 (50.0%) | - |
| | <50% | 44 (50.0%) | 3.29 (1.96-5.50, p < 0.001) |
| **hsa.miR.625.5p** | Low | 33 (37.5%) | - |
| | High | 55 (62.5%) | 2.04 (1.24-3.35, p= 0.005) |
