# Supplementary material for: Large-Scale Profiling of Extracellular Vesicles Identified miR-625-5p as a Novel Biomarker of Immunotherapy Response in Advanced Non-Small-Cell Lung Cancer Patients
Source: Cancers (Basel). 2022 May 14;14(10):2435. doi: 10.3390/cancers14102435 (PMC9139420; doi:10.3390/cancers14102435)
Supplement: Supplementary file 1 [file cancers-14-02435-s001.zip › Supplementary table S4.pdf]

Clinical Information	p value	Spearman r	Confidence intervals of rs
Adrenal Metastasis	0.97433246	-0.003479614	-0.2188 to 0.2121
Age	0.71007269	0.04018819	-0.1768 to 0.2534
Bone Metastasis	0.54031029	0.066150351	-0.1515 to 0.2776
Brain Metastasis	0.14378505	0.157109517	-0.06038 to 0.3604
Hystology	0.67627126	-0.045134342	-0.2581 to 0.1720
Liver Metastasis	0.04688526	0.212463157	-0.003123 to 0.4092
Lung Metastasis	0.38984522	0.092794242	-0.1252 to 0.3022
Node Metastasis	0.55604102	0.063604891	-0.1539 to 0.2753
Other Metastasis	0.87575026	0.01690847	-0.1993 to 0.2315
PDL1Staining	0.00827086	-0.279863462	-0.4671 to -0.06855
Pleural Effusion	0.28186678	0.115987117	-0.1020 to 0.3234
SoftTissue Metastasis	0.80551843	0.026622247	-0.1899 to 0.2407
Therapy Line	0.09359564	0.179850726	-0.03703 to 0.3805
TumorTargetsizeBaseline	0.750219	0.039319731	-0.2079 to 0.2818
