# Supplementary material for: Large-Scale Profiling of Extracellular Vesicles Identified miR-625-5p as a Novel Biomarker of Immunotherapy Response in Advanced Non-Small-Cell Lung Cancer Patients
Source: Cancers (Basel). 2022 May 14;14(10):2435. doi: 10.3390/cancers14102435 (PMC9139420; doi:10.3390/cancers14102435)
Supplement: Supplementary file 1 [file cancers-14-02435-s001.zip › Supplementary table S5.pdf]

Clinical Information	OR	Lower 95%	Upper 95%	Original P value
Age >70 yy	0.836	0.323	2.165	0.712
Bone Met	0.616	0.223	1.701	0.35
Brain Met	0.459	0.120	1.751	0.255
Current Smoker	0.347	0.072	1.661	0.185
Current Smoker	2.143	0.775	5.924	0.142
ECOG 0-1	0.694	0.267	1.801	0.452
EGFRmut	1.106	0.582	2.099	0.759
Histology Adenocarcinoma	1.600	0.521	4.914	0.412
Liver Met	0.702	0.207	2.384	0.57
Lung Met	0.945	0.365	2.449	0.908
Node Met	0.988	0.312	3.133	0.984
Other Site Met	0.250	0.030	2.071	0.199
Pleural Effusion	0.347	0.072	1.661	0.185
Sex Male	0.774	0.282	2.128	0.62
SoftTissue Met	0.545	0.060	4.932	0.59
Surgery on Primary Cancer	1.106	0.582	2.099	0.759
TumorBurden	0.702	0.207	2.384	0.57
