# Supplementary material for: Large-Scale Profiling of Extracellular Vesicles Identified miR-625-5p as a Novel Biomarker of Immunotherapy Response in Advanced Non-Small-Cell Lung Cancer Patients
Source: Cancers (Basel). 2022 May 14;14(10):2435. doi: 10.3390/cancers14102435 (PMC9139420; doi:10.3390/cancers14102435)
Supplement: Supplementary file 1 [file cancers-14-02435-s001.zip › Supplementary table S6.pdf]

Clinical Information	HR	Lower 95%	Upper 95%	Original P value
Adrenal Met	0.9577	0.4955	1.8509	0.8977
Age >70 yy	0.8043	0.4936	1.3105	0.3819
Bone Met	0.8698	0.4788	1.5802	0.6471
Brain Met	1.9885	0.8491	4.6571	0.1134
Current Smoker	0.9147	0.5243	1.5957	0.7535
EGFRmut	1.8161	0.6522	5.0573	0.2535
Histology Adenocarcinoma	0.6930	0.4115	1.1668	0.1677
Lung Met	0.8698	0.4788	1.5802	0.6471
Node Met	0.9232	0.5094	1.6732	0.7923
Other Site Met	1.2385	0.5849	2.6226	0.5763
Pleural Effusion	1.0631	0.6514	1.7352	0.8065
Sex Male	1.1197	0.6480	1.9349	0.6854
SoftTissue Met	0.7858	0.2835	2.1780	0.6431
Surgery on Primary Cancer	0.9553	0.6326	1.4427	0.8279
