# Supplementary figures and images for: Large-Scale Profiling of Extracellular Vesicles Identified miR-625-5p as a Novel Biomarker of Immunotherapy Response in Advanced Non-Small-Cell Lung Cancer Patients
Source: Cancers (Basel). 2022 May 14;14(10):2435. doi: 10.3390/cancers14102435 (PMC9139420; doi:10.3390/cancers14102435)

**A**

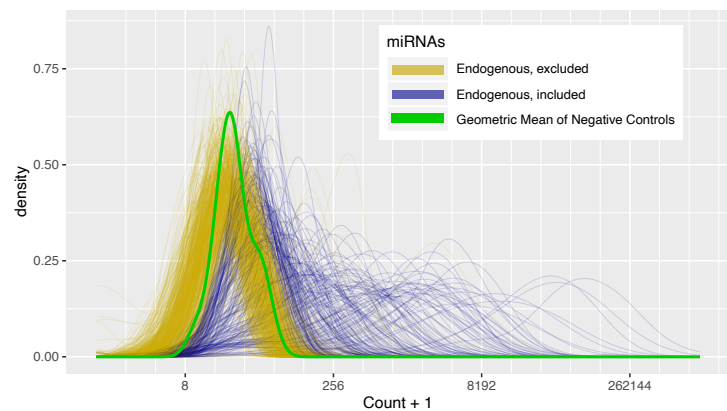

**B**

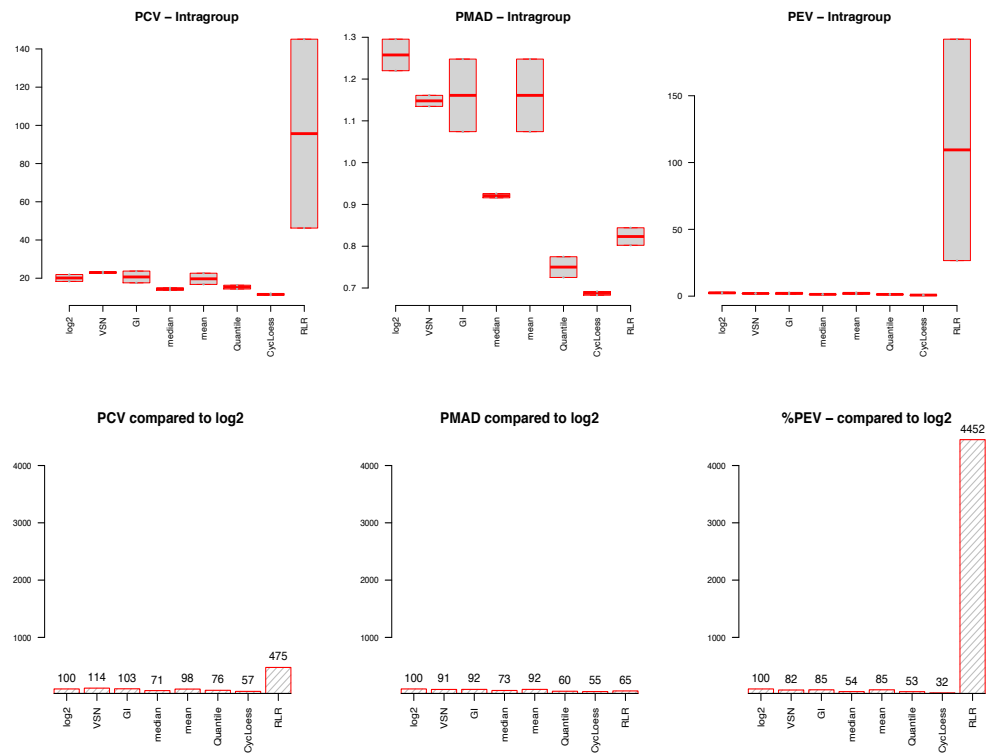

**C**

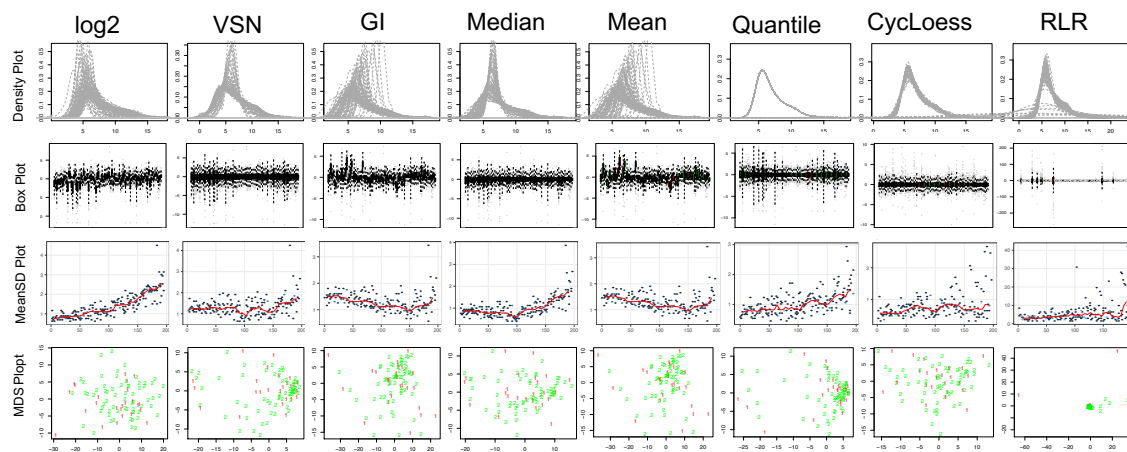

Supplement: Supplementary file 1 [file cancers-14-02435-s001.zip › Supplementary Figure S1.pdf]

A

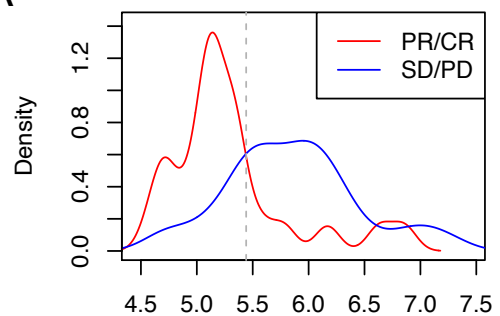

hsa-miR-625-5p Normalized Counts

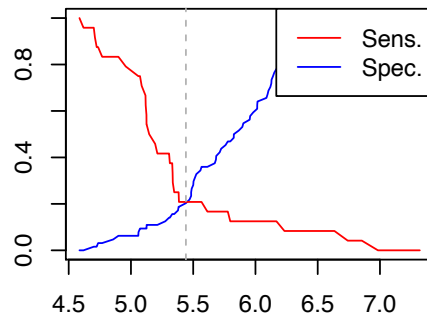

hsa-miR-625-5p Normalized Counts

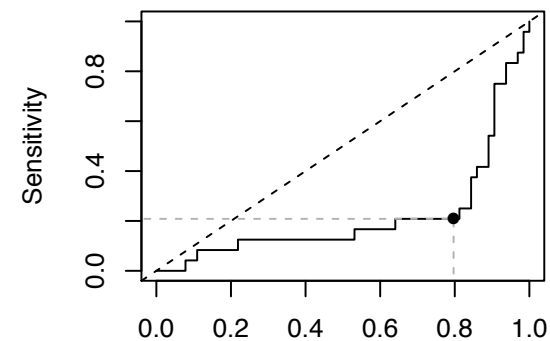

1-Specificity

B

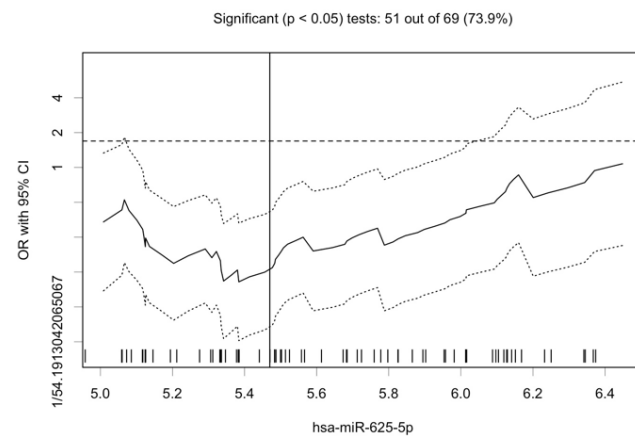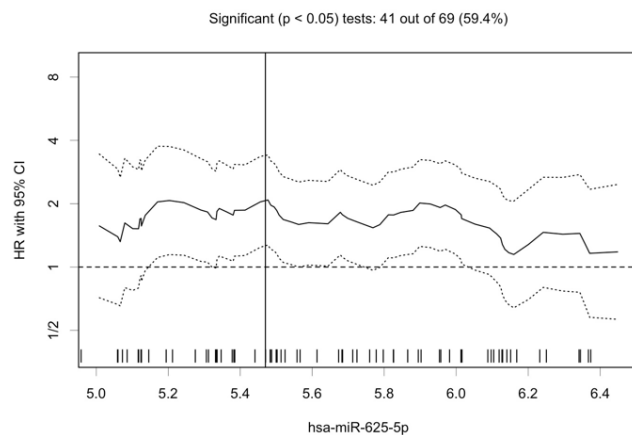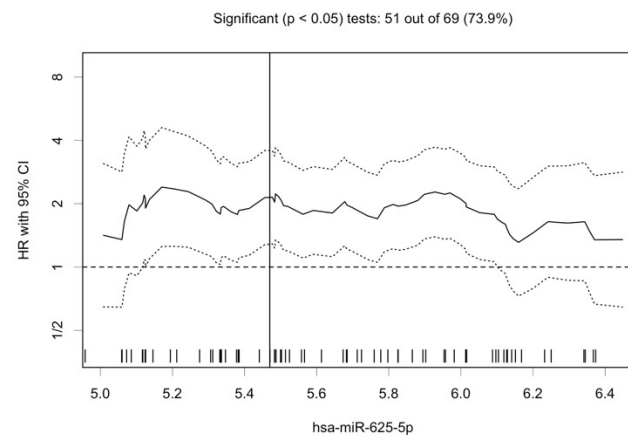

Supplement: Supplementary file 1 [file cancers-14-02435-s001.zip › Supplementary Figure S2.pdf]

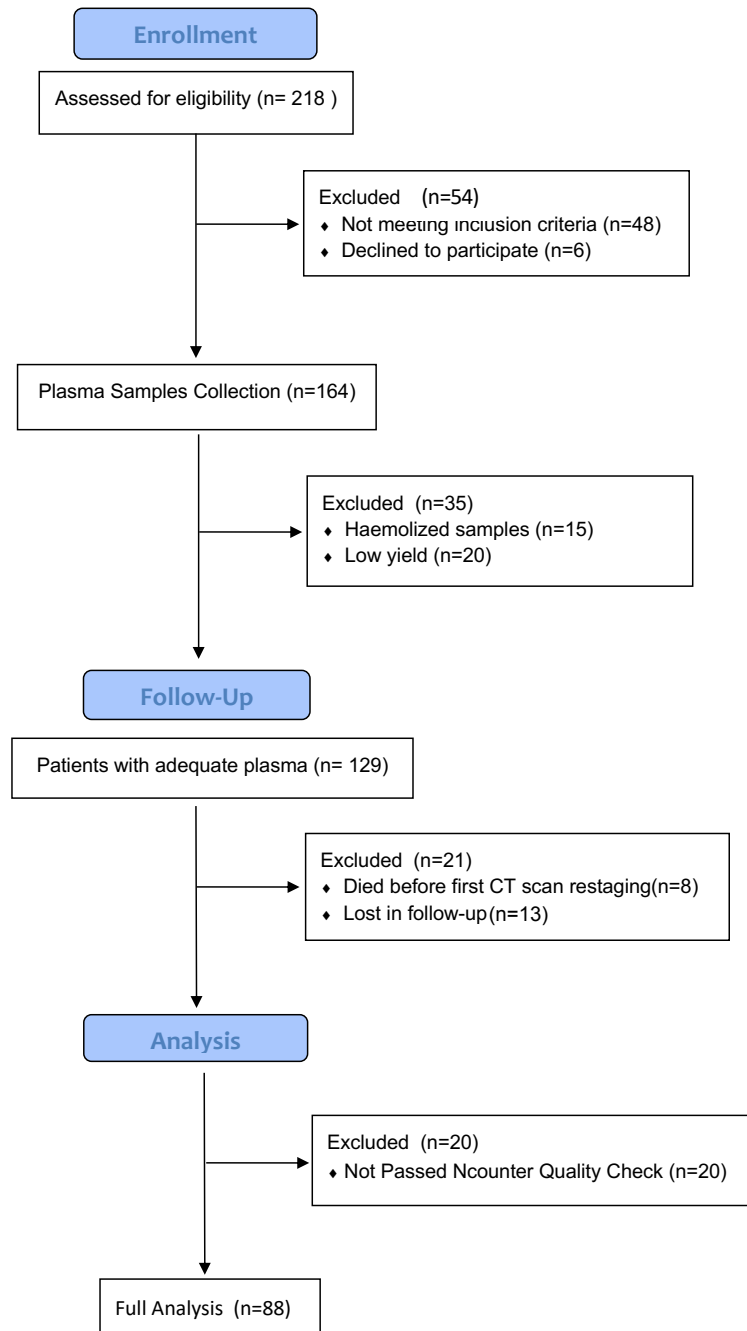

Supplement: Supplementary file 1 [file cancers-14-02435-s001.zip › Supplementary Figure S3.pdf]

2DLL

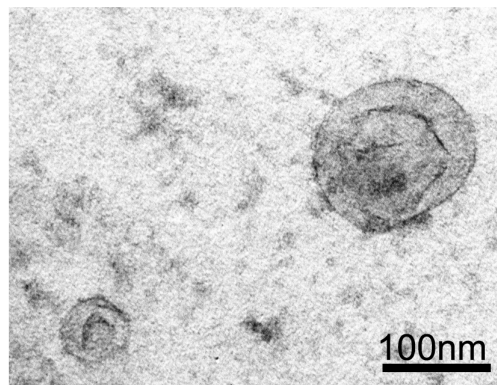

19DGM

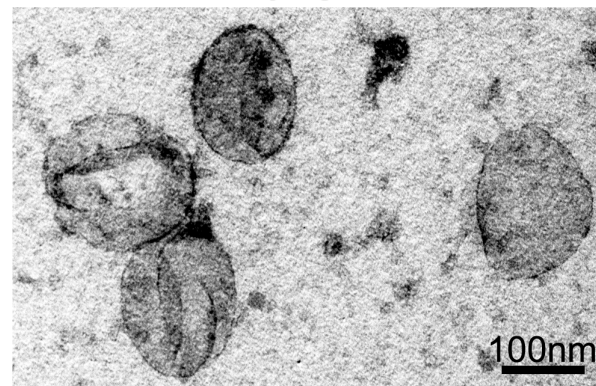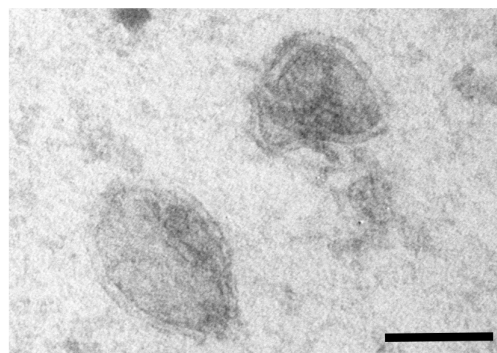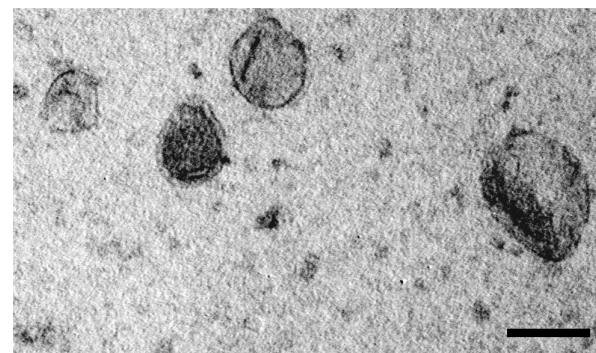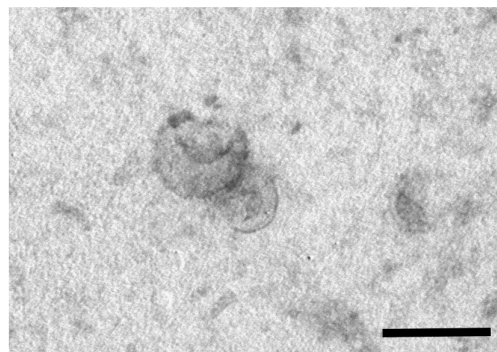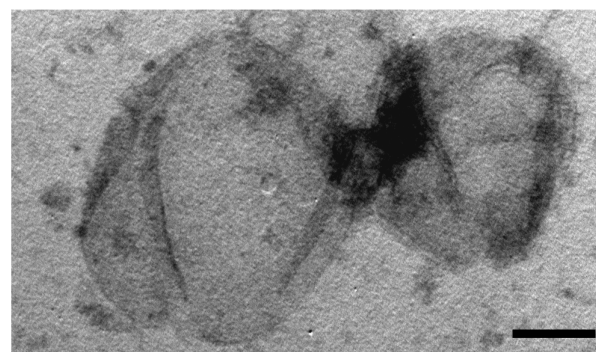

Supplement: Supplementary file 1 [file cancers-14-02435-s001.zip › Supplementary Figure S4.pdf]

**A**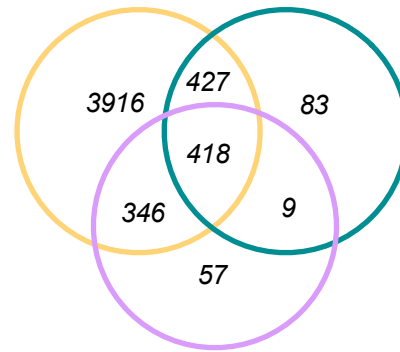**B**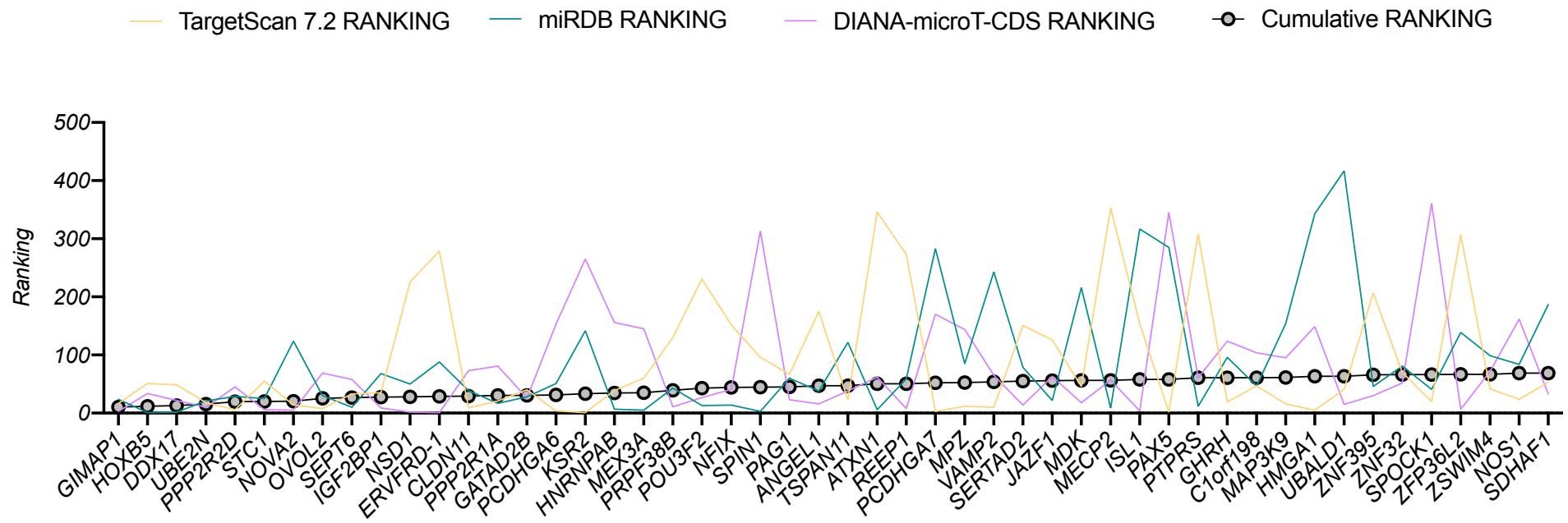

Supplement: Supplementary file 1 [file cancers-14-02435-s001.zip › Supplementary Figure S5.pdf]

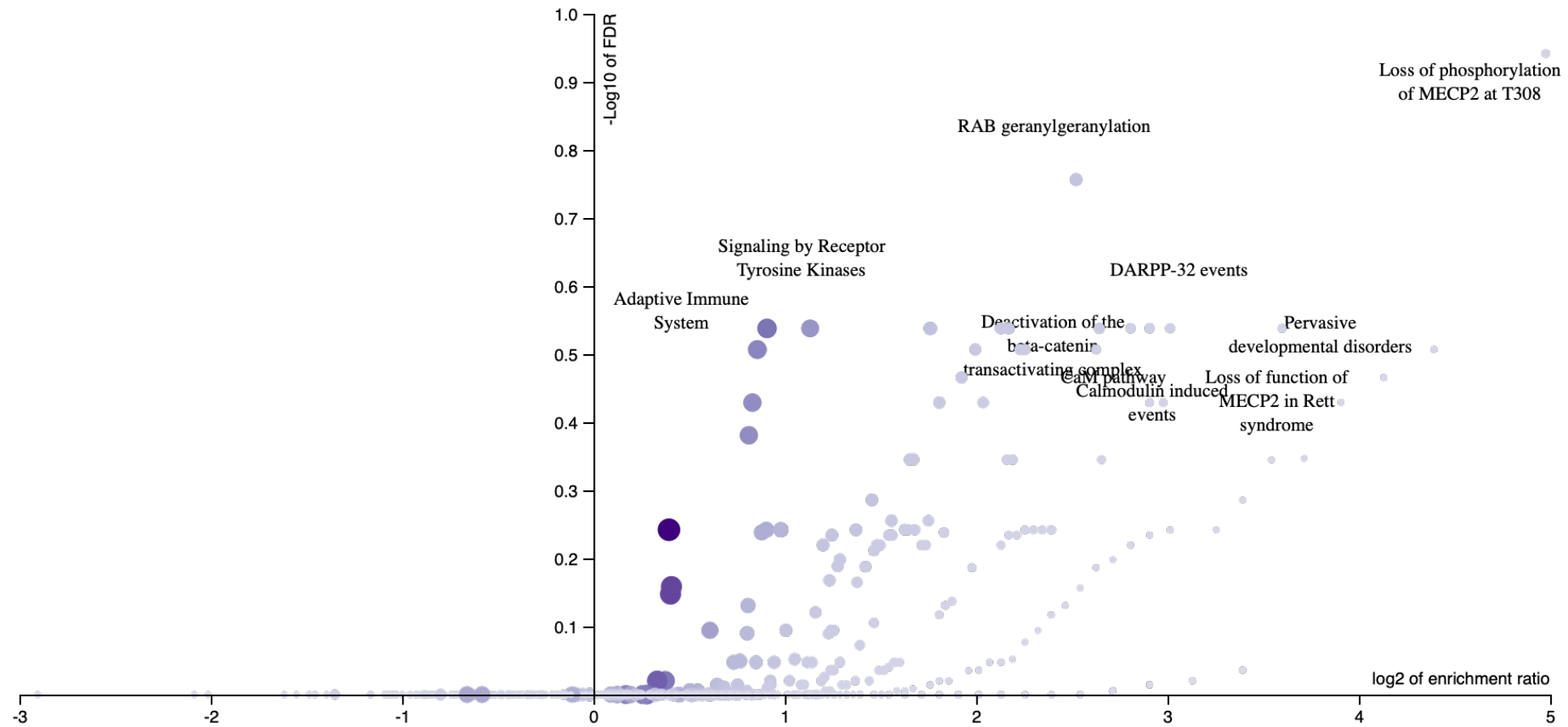[illegible]

Supplement: Supplementary file 1 [file cancers-14-02435-s001.zip › Supplementary Figure S6.pdf]
